# A Comparative* In Vivo* Study on Three Treatment Approaches to Applying Topical Botulinum Toxin A for Crow's Feet

**DOI:** 10.1155/2018/6235742

**Published:** 2018-07-03

**Authors:** Yan Cao, Jian-ping Yang, Xiao-gang Zhu, Jie Zhu, Hong-qin Chang, Sheng-hua Guo, Dan Luo, Bing-rong Zhou

**Affiliations:** ^1^Department of Dermatology, The Affiliated Changzhou No. 2 People's Hospital of Nanjing Medical University, Changzhou 213000, China; ^2^Department of Orthopaedics, Changzhou Traditional Chinese Medical Hospital Affiliated to Nanjing University of Traditional Chinese Medicine, 25 Heping North Road, Changzhou 213000, China; ^3^Department of Dermatology, The First Affiliated Hospital of Nanjing University of TCM, Nanjing, Jiangsu 210029, China; ^4^Department of Dermatology, The First Affiliated Hospital of Nanjing Medical University, Nanjing 210029, China

## Abstract

**Objective:**

To evaluate the efficacy and safety of three treatment approaches to applying Botulinum Toxin Type A (BoNTA) for crow's Feet.

**Methods:**

Thirty female subjects with moderate-to-severe crow's feet were included in this comparative* in vivo *study. They were randomly divided into three groups, including the local intramuscular, intradermal microdroplet injection, and nanomicroneedle delivered with BoNTA therapy group. After one session, evaluations were done at the time points of weeks one, four, and twelve after the treatment. The assessments included subjective satisfaction, blinded clinical assessment, and the biophysical parameters (skin collagen content, elasticity, hydration, and sebum contents).

**Results:**

For dynamic wrinkles, intramuscular injection and intradermal microdroplets injection were more effective than nanomicroneedles. For static wrinkles, nanomicroneedles and intradermal microdroplets injection were more effective. However, the intramuscular injection had no significant effect on static wrinkles. At one week and four weeks after the treatment, the skin elasticity, collagen content, and hydration of nanomicroneedle group and intradermal microdroplet group increased more significantly than those of the intramuscular injection group; at twelve weeks after the treatment, the skin elasticity, collagen content, and hydration of intradermal microdroplet group were higher than those of other two groups. However we observed no statistically significant difference in sebum content between the three groups before and after the treatment.

**Conclusion:**

BoNTA delivered through nanomicroneedles and intradermal microdroplets injection can effectively treat crow's feet. This trial is registered with [2016]KY018-01, registered 16 Feb 2016.

## 1. Introduction

**Figure 1:**
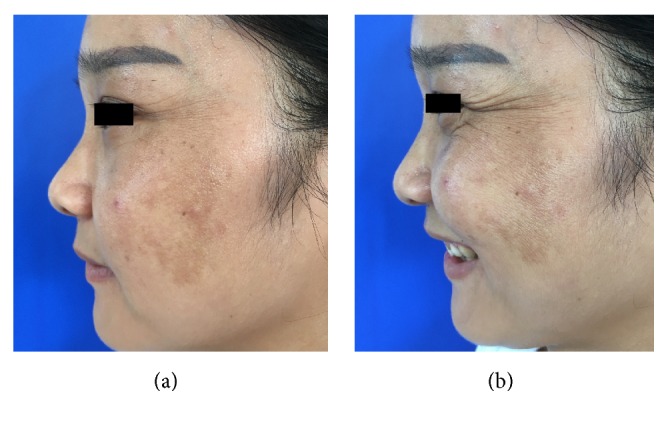
Representative clinical photographs of the crow's feet's classification: (a) one with the face at rest (static wrinkles) and (b) one with the face at maximum smile (dynamic wrinkles).

The periorbital region is an area most vulnerable to aging, usually attacked by crow's feet or lateral canthal lines. Rhytides are caused by the gradual loss and disorganization of underlying collagen and elastin fibers that support the skin [1]. Repeated facial expressions [2], ultraviolet light exposure [1], and smoking [3] can decrease the skin elasticity and form wrinkles. Crow's feet are accentuated as orbicularis oculi muscles contract to control the movements of the eyelids [1]. Two scales are available to determine the clinical severity of crow's feet [4]: one scale for facial wrinkles at rest (static wrinkles) and one for facial wrinkles at maximum smile (dynamic wrinkles) (Figure 1).

Botulinum Toxin Type A (BoNTA) is a neurotoxin protein secreted by clostridium botulinum, which is an anaerobic, Gram-positive bacterium. It blocks the transmission of nerve impulses to the targeted muscle by selectively preventing the release of the neurotransmitter acetylcholine at the neuromuscular junction, a process that can temporarily prevent muscle contraction [5]. The ability of BoNTA to alleviate crow's feet was first described in 1993 [6]. Since that time, it has been widely used in the cosmetic field to reduce wrinkles and rejuvenate the skin [7]. BoNTA can be used in the perioral or other parts of the head and the neck, either singly or combined with other cosmetic operations [8]. BoNTA has been conventionally administered through local intramuscular or intradermal microdroplets injection and proven effective in reversing facial wrinkles [9]. Multiple injections can lead to complications such as pain, erythema, bruising, and potential infection; also, misplaced injections can pose undesired effects on nontarget areas, such as eyelid brow ptosis and partial lip ptosis [10].

An assumption is that large-size molecules hamper the penetration through the skin barrier. Interestingly, a delivery method using a roller studded with fine needles, termed as microneedle therapy, has been invented and used successfully in improving scars and wrinkles and rejuvenating faces through collagen induction [11]. In addition, transdermal drug delivery becomes easy by making multiple mechanical holes in the skin [12]. Nanomicroneedle therapy, a modified microneedle therapy, has come into use with more advantages, like convenience, fast, and painlessness. Compared with the traditional microneedle therapy [13], nanomicroneedle therapy makes skin barrier functions recover more quickly and transdermal drug delivery more efficient. We hypothesize that nanomicroneedle delivered with BoNTA therapy could facilitate the transcutaneous penetration of BoNTA and collagen remodelling, because nanomicroneedles itself could induce collagen production in addition to making holes that improve the transdermal delivery of BoNTA. Therefore, our research group performed this prospective, randomized, and controlled* in vivo *study to compare the efficacy of three approaches to treat crow's feet: the local intramuscular, intradermal microdroplet injection, and nanomicroneedle delivered with BoNTA therapy.

## 2. Subjects and Methods

### 2.1. Subjects

A total of 30 females (aged 30 to 51, mean of 39.9, weighing 55 ± 3 kg) with moderate-to-severe crow's feet wrinkles were enrolled. All subjects provided written informed consent, and this study was approved by the Institutional Review Board of the Affiliated Changzhou No. 2 People's Hospital of Nanjing Medical University. All subjects' conditions were classified into grade 3 and grade 4 according to the Global Assessment of Lateral Canthal Lines scale (the IGA-LCL [14] 5-point scale): 0: no wrinkles, 1: minimal wrinkles, with/without minimal etching within a radius of 1.5 cm radius surrounding the lateral canthus, 2: mild wrinkles, with minimal etching within a radius of 1.5-2.5 cm surrounding the lateral canthus, 3: moderately deep wrinkles with moderate etching within a radius of 1.5-2.5 cm surrounding the lateral canthus, and 4: severe and long wrinkles, deeply etched in a radius of ≥2.5 cm surrounding the lateral canthus).

Exclusion criteria included any use of BoNTA or any resurfacing procedure on crow's feet during the six months before the study, hypersensitivity to injected materials, pregnant females, and history of keloids or abnormal scarring.

### 2.2. Methods

#### 2.2.1. Applied Substances

The BoNTA used in this study was the oldest biosimilar BoNTA, first licensed in 1997 and also known as “Hengli” toxin [15]. The BoNTA powder (HENGLI® Lanzhou Institute of Biological Products Co., Ltd., Lanzhou, Gansu, China) was dissolved into injectable saline solution. Each vial of 50 LD50 units of BoNTA was reconstituted with 1.0 mL preservative free sterile saline (0.9% isotonic sodium chloride) to drop the concentration to 1U per 0.02 mL.

#### 2.2.2. Groups

Thirty subjects were randomly divided into three groups.

*Intramuscular Injection Group*. Ten subjects received intramuscular injections of BoNTA into three defined points in the crow's feet area on both sides of the face. The total dose was 14U (7U/side). The first injection was made 1.5 cm lateral to the outer canthus (dose of 3U), and the other two injection points (2U per injection) were made 1 cm above and below the primary injection point [16].

*Intradermal Microdroplet Injection Group. *Ten subjects received intradermal microdroplets of BoNTA into seven injection points in the crow's feet area on both sides of the face (1U/0.02 mL per point]. The total dose was 14U (7U/side). The intradermal microdroplet technique targeted the outermost muscle fibers in the dermis. To achieve this, a needle was advanced slowly with a bevel parallel to the skin and the plunger was pressed until a small bleb appeared from the skin.

*Nanomicroneedle Therapy Group. *The nanomicroneedle consists of a rechargeable hand-piece with disposable needles driven by a motor. The nanomicroneedles are composed of an array of nanochips attached to the plastic crystal head (provided by Suzhou Natong Bio-Nano Technology Co., Ltd., Suzhou City, China).

Specifications for 0.25 mm nanotips [13] were as follows: needle length 0.25 mm, diameter 120 nm, nanochip length 3 mm, width 3 mm, thickness 0.4 mm, quantity 36; array height 0.25 mm; taper 40°.

The device was applied three times back and forth, and then 1.4 mL of BoNTA solution (diluted in saline, concentration at 5U/mL) was immediately topically applied on each side for 20 min.

### 2.3. Outcome Assessment

Efficacy was assessed by both investigators and subjects. The primary endpoint was an improvement in physician-rated crow's feet severity both at rest and maximum expression. The outcome was assessed based on the subjective satisfaction, scores calculated by blinded investigators (using standardized photography), and biophysical results. The assessment was conducted at the time point of baseline, one, four, and twelve weeks after the treatment.

#### 2.3.1. Subjective Satisfaction

Each subject completed a self-assessment questionnaire and rated their condition with a score from 0 (aggravated) to 4 (much improved). After the treatment, all the subjects graded their intraprocedural pain on a 10 cm visual analog scale (VAS), with the end points designated as 0 (no pain) and 10 (the intolerable pain). The investigator subjectively graded the edema severity after treatment on a scale from 0 to 4 (0: absent, 1: trace, 2: slight, 3: moderate, and 4: prominent). The duration of erythema and crusting was investigated through interviews. Any adverse events and complications were recorded during the treatment and the follow-up.

#### 2.3.2. Blinded Clinical Assessment

Standardized photographs were obtained at baseline and weeks one, four, and twelve after the treatment. Standardized close-up photographs were taken using a high-resolution digital camera (Canon EOS-40D, Canon Corp, Tokyo, Japan). Two dermatologists who were blind to the treatment group evaluated the serial photographs independently and performed clinical assessments on the crow's feet using the IGA-LCL scale.

#### 2.3.3. Biophysical Evaluation

The parameters (skin elasticity, hydration, collagen content, and skin sebum contents) were measured using CBS-Medicai skin analysis system (CBS®, WUHAN BOSEELECTRONIC CO., LTD, Wuhan, China). The CBS skin analysis system is based on the principle of spectrum color gradation recognition and texture scanning, supplemented by statistical foundations, to obtain data and results of the corresponding test items [17]. All measurements were taken after subjects had passed an adaptation period of at least 20 minutes in an air-conditioned room at 22–25°C, 50% humidity.

### 2.4. Statistical Analysis

The results were analyzed with the single factor variance analysis using SPSS 15.0 software (SPSS, Inc., Chicago, IL). *P* < 0.05 was considered statistically significant.

## 3. Results

### 3.1. Clinical Assessment

**Figure 2:**
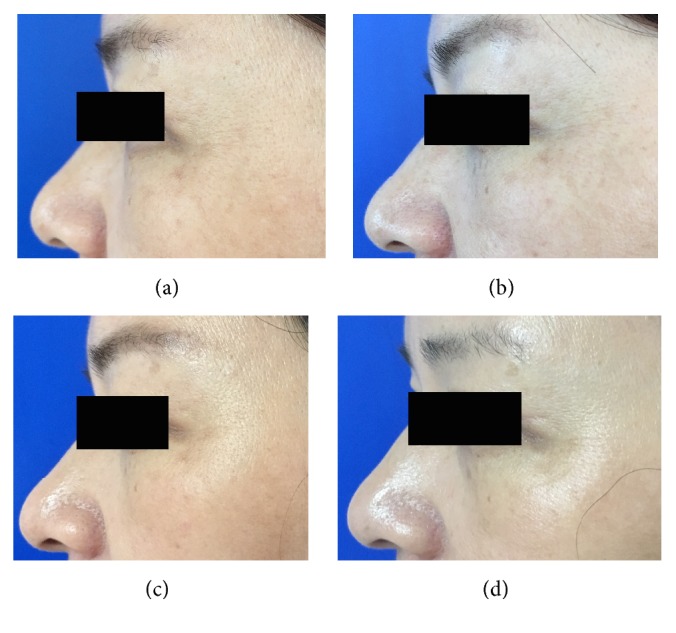
Representative clinical photographs of intramuscular injections technique group (crow's feet static wrinkles): (a) baseline, (b) 1 week after treatment, (c) 4 weeks after treatment, and (d) 12 weeks after treatment.

**Figure 3:**
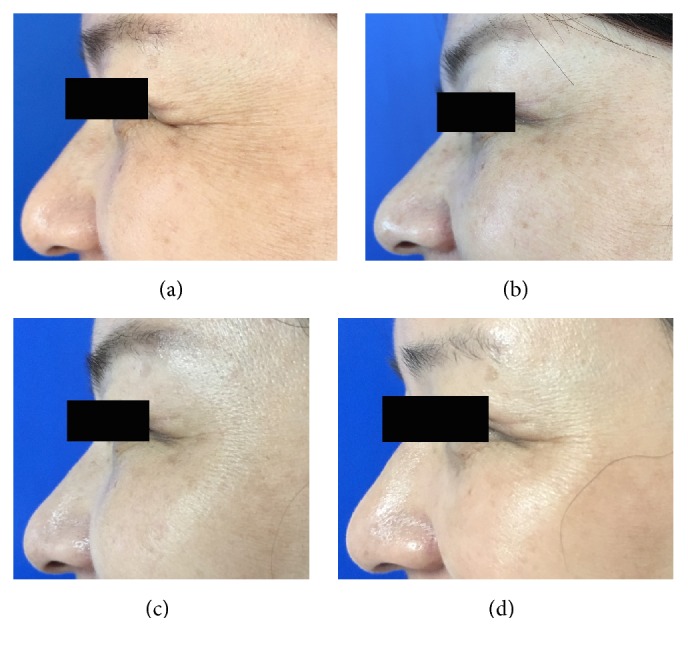
Representative clinical photographs of intramuscular injections technique group (crow's feet dynamic wrinkles): (a) baseline, (b) 1 week after treatment, (c) 4 weeks after treatment, and (d) 12 weeks after treatment.

**Figure 4:**
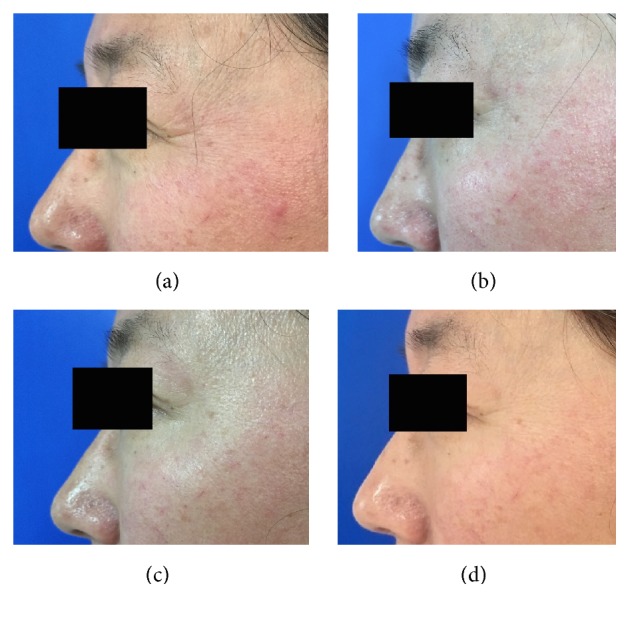
Representative clinical photographs of intradermal microdroplet technique group (crow's feet static wrinkles): (a) baseline, (b) 1 week after treatment, (c) 4 weeks after treatment, and (d) 12 weeks after treatment.

**Figure 5:**
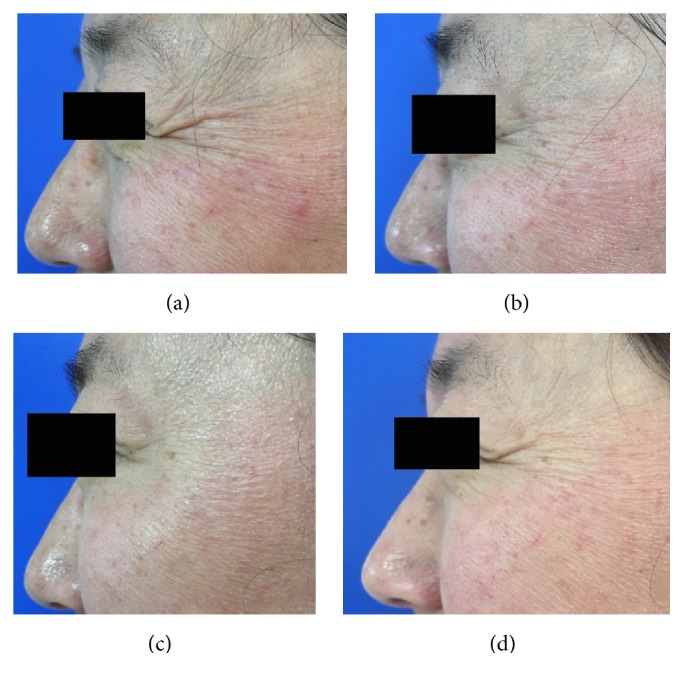
Representative clinical photographs of intradermal microdroplet technique group (crow's feet dynamic wrinkles): (a) baseline, (b) 1 week after treatment, (c) 4 weeks after treatment, and (d) 12 weeks after treatment.

**Figure 6:**
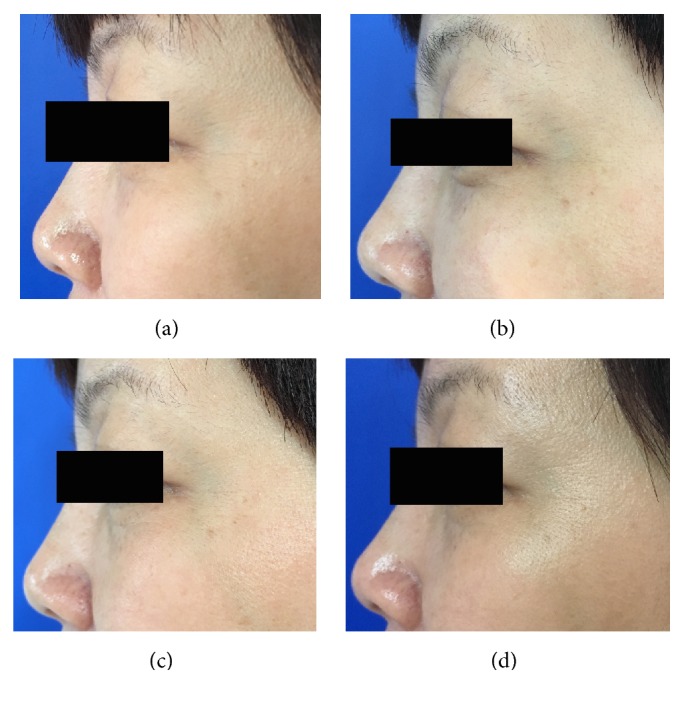
Representative clinical photographs of nanomicroneedles group (crow's feet static wrinkles): (a) baseline, (b) 1 week after treatment, (c) 4 weeks after treatment, and (d) 12 weeks after treatment.

**Figure 7:**
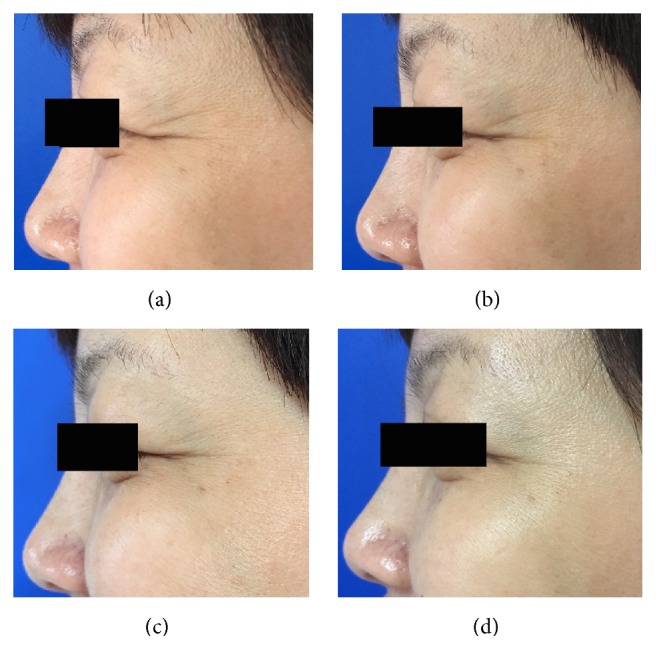
Representative clinical photographs of nanomicroneedles group (crow's feet dynamic wrinkles): (a) baseline, (b) 1 week after treatment, (c) 4 weeks after treatment, and (d) 12 weeks after treatment.

When the crow's feet severity was assessed in a dynamic state, all the three groups showed a statistically significant difference from baseline to one, four, or twelve weeks after the treatment. However, the nanomicroneedle group showed lower efficacy than the intramuscular injection group and the intradermal microdroplet injection group (Table 1, Figures 2–7).

When the crow's feet severity was assessed in a static condition, a statistically significant difference was detected from baseline to one, four, or twelve weeks after the treatment on both the nanomicroneedle group and the intradermal microdroplet group; however, no statistically significant difference was detected on the intramuscular injection group before and after treatment. The difference at weeks one, four, and twelve was not statistically significant between the nanomicroneedle group and the intradermal microdroplet group (Table 2, Figures 2–7).

### 3.2. Subjective Satisfaction Scale

The subjective satisfaction at week one, four, or twelve after the treatment showed no statistically significant difference between the three groups (Table 3).

### 3.3. Adverse Reactions

The pain scores of the three groups showed statistically significant differences. The nanomicroneedle group showed lower pain score than the intramuscular injections group and the intradermal microdroplet group.

The intramuscular injections group showed severer erythema and edema than the nanomicroneedle group and intradermal microdroplet group (Table 4).

Throughout the study, no serious or persistent adverse effects occurred and no one withdrew from the study because of adverse events. No potential infection or no brow ptosis was observed.

### 3.4. Biophysical Analysis

#### 3.4.1. Skin Elasticity

One week after the treatment, the skin elasticity of the three groups increased, but showed no significant between-group difference. Four weeks after the treatment, the skin elasticity in the nanomicroneedle group and the intradermal microdroplet group increased and became higher than that in the intramuscular injection group; then twelve weeks after the treatment, the skin elasticity in the intradermal microdroplet group was higher than that in the intramuscular injection group, and that in the intramuscular injection group was higher than that in the nanomicroneedle group; however, we observed that the skin elasticity of all three groups insistently increased at weeks one, four, and twelve after the treatment (Table 5).

#### 3.4.2. Skin Hydration

Skin hydration is an index to evaluate the function of skin barrier. As shown in Table 6, skin hydration of the three groups increased significantly from baseline to week one, four, or twelve after the treatment. One week and four weeks after the treatment, the skin hydration in the nanomicroneedle group and the intradermal microdroplet group increased and became higher than that of the intramuscular injection group; then twelve weeks after the treatment, the skin hydration in the intradermal microdroplet group was higher than that of the intramuscular injection group and the nanomicroneedle group.

#### 3.4.3. Skin Collagen Content

It is well known that the collagen in the dermis is mainly composed of collagen I(80%~85%) and collagen III(10%~15%), which are responsible for the elasticity and intensity of skin and for producing a healthier skin texture [18]. The CBS skin analysis system used the 3D Negative Technique to analyze the collagen fiber. The signals in the dermis are mainly produced by collagen I fibers, attributing to the collagen I fibers structure and their arrangement in tissues. Because collagen I fibers from the reticular dermis are thicker and loosely arranged and they are interwoven into a mesh, parallel to the skin surface. These signals of collagen I fibers are more easily visible. Although collagen III fibers in the papillae are more superficial, their signals are easily disturbed by the scattered LED light and become vague. As shown in Table 7, one week after the treatment, the skin collagen I contents of the three groups were all higher than those prior to the treatment, but showed no statistically significant between-group differences. Four weeks after the treatment, the skin collagen I contents in the nanomicroneedle group and the intradermal microdroplet group increased and were higher than that in the intramuscular injection group; then twelve weeks after the treatment, the skin collagen I content in the intradermal microdroplet group was higher than that in the intramuscular injection group, and that in the intramuscular injection group was higher than that in nanomicroneedle group. However, we observed the skin collagen I content of the three groups all continuously increased at weeks one, four, and twelve after the treatment.

#### 3.4.4. Skin Sebum Content

Sebum is a sticky liquid mixture of nonpolar lipids. The composition of sebum is relatively constant, and its changes may entail some skin diseases. Production of sebum depends on individual characteristics, environmental factors, and the density, location, and activity of sebaceous glands.

No statistically significant difference was detected from the baseline to week one, four, or twelve after the treatment among the three groups (Table 8).

## 4. Discussion

In our study, when treating dynamic wrinkles, intramuscular injection and intradermal microdroplets group showed more significant effectiveness than the nanomicroneedle group. The underlying mechanism is that nanomicroneedle can create microscopic columns in the epidermis and dermis with the expectation that this would permit molecules of BoNTA to reach the superficial orbicularis oculi in the lower dermis. And the transcutaneous penetration doses of BoNTA by nanomicroneedle may be partially reduced.

In our study, we found that half of the patients saw the improved condition of crow's feet wrinkles within twenty-four hours after treatment, with a median onset time of twelve to twenty hours, and all the patients reported the improvement six days after the treatment. These results are consistent with those of the previous studies [19, 20]. In general, some patients are aware of an improvement in wrinkles that seems to occur 3 to 6 months after treatment [21]. In our study we found that most patients maintained a near full effect of the toxin 2 months after treatment; by 3 months the effect had declined to some patients, but almost all patients in nanomicroneedle group.

When treating static wrinkles, nanomicroneedle therapy and intradermal microdroplets technique showed effectiveness at weeks one, four, and twelve after the treatment. However, the intramuscular injection had no significant effect on static wrinkles. We also observed that the nanomicroneedle therapy and the intradermal microdroplet technique can improve skin texture in the treatment area. The performance of the skin in the treatment area is more delicate and smooth. Besides that, we observed that the skin elasticity and collagen I content of all three groups increased at weeks one, four, and twelve after the treatment. Four weeks after the treatment, the skin elasticity and collagen I content of the nanomicroneedle group and the intradermal microdroplet group increased more significantly than the intramuscular injection group; twelve weeks after the treatment, the intradermal microdroplet group had higher elasticity and collagen I content than the intramuscular injection group, and that in the intramuscular injection group was higher than that in the nanomicroneedle group.

Our preliminary study [22] demonstrated for the first time that BoNTA has positive effect on UVB-SIPS HDFs in vitro by increasing collagen production, inhibiting collagen degradation, and stimulating cell proliferation via decreasing senescence related proteins. This indicates that BoNTA can play an antiphotoaging role in anti-UVB-induced premature senescence. Now our results in the present* in vivo *study further confirmed the antiphotoaging role of BoNTA by improving skin collagen I production and elasticity, especially in nanomicroneedle group and intradermal microdroplet group. However, we found that this effect lasted less than three months in the nanomicroneedle group, shorter than that in the intradermal microdroplet group.

Skin hydration is also an index to evaluate the function of skin barrier. Skin hydration of the three groups showed a statistically significant increase at week one, four, or twelve after the treatment. One week and four weeks after the treatment, the skin hydration in the nanomicroneedle group and the intradermal microdroplet group increased more significantly than that in the intramuscular injection group; then twelve weeks after the treatment, the skin hydration in the intradermal microdroplet group was higher than that in the intramuscular injection group and nanomicroneedle group. It can be inferred that better skin barrier function can be achieved by BoNTA topical treatment, especially in nanomicroneedle group and the intradermal microdroplet group.

Sebum is a sticky liquid mixture of nonpolar lipids, with a relatively stable composition. The production of sebum is dynamically affected by individual characteristics and environmental factors, like density, location, and activity of sebaceous glands mainly regulated by androgen. It has been reported [23–25] that intradermal or intramuscular injection of BoNTA may be an effective treatment to reduce sebum production of patients with oily skin. However in our study we observed that no statistically significant difference of sebum content was detected before and after the treatment in the three groups. The influence factors may be dietary, endocrinic, and minor differences of temperature and humidity during sebum measurement. The underlying mechanisms deserve a large sample study.

As to erythema and edema being complicated with the treatment, in our study we found that the Botulinum Toxin A-induced swelling in all subjects subsided within 48-72 hours. the intramuscular injection group showed a higher incidence than the nanomicroneedle group and the intradermal microdroplet group. The pain score of the nanomicroneedle group was lower than that of the intramuscular injection group and intradermal microdroplet group.

Comparing with intramuscular injection technique, the intradermal microdroplet technique prevents diffusion of BoNTA into deeper muscles, which can lead to a “frozen” appearance. It is proposed that the microdroplet injections can smooth and tighten the skin by inducing bulk atrophy of the sweat and sebaceous glands, weakening the superficial muscle fibers in the skin, and thereafter reducing the pulling and tethering force of the facial muscles that form fine lines and wrinkles. We found that microdroplet injections technique could reduce dynamic wrinkles and static wrinkles and improve skin barrier function. Its treatment effects usually last for 3 to 4 months.

BoNTA, a type of large molecule, has a low cutaneous bioavailability—simply applying its solution to intact skin is not effective [26]. Combinations of BoNTA to correct wrinkles can achieve the same (or better) results compared to an individual treatment approach, potentially in a shorter time frame and convenience to the patient [27, 28]. The treatment also results in very high (>90%) patient satisfaction scores [28–30]. Two studies have used a fractional ablative laser to create microscopic columns in the epidermis and dermis that permitted BoNTA molecules to reach the superficial orbicularis oculi in the lower dermis [31, 32]. Although the fractional ablative laser combination with BoNTA was successful, its operation is more complicated and cost is higher than injections [32]. The nanomicroneedle therapy, a newly developed method, is convenient, quick, painless, and suitable for transdermal drug delivery. It can open tiny holes in the skin facilitating the transcutaneous penetration of BoNTA [12]; it might have good effects on collagen remodelling, because the nanomicroneedle itself induces collagen production in addition to making holes that improve transdermal delivery of BoNTA. It could improve skin barrier function and reduce static wrinkles effectively, but it has week effect on dynamic wrinkles, and the time maintaining the effect is shorter than injection technique.

One major limitation of this study is its small sample size. Larger sample randomized controlled clinical trials are needed to verify the results of this study. To the best of our knowledge, our study is the first to report the efficacy of the nanomicroneedle therapy delivered with BoNTA in treating crow's feet and compare it with conventional injections. We conclude that BoNTA delivered through nanomicroneedle and intradermal microdroplets injection can effectively treat crow's feet. Its exact mechanism of action and how to optimize its clinical effects are still worth further researches.

## Figures and Tables

**Table 1:** Comparison of clinical effect on dynamic wrinkles before and after treatment ($\overset{-}{x}\pm S$).

Group	Baseline	1 week	4 weeks	12 weeks
intramuscular injections	3.60 ± 0.50	1.00 ± 0.46^*∗*c^	0.50 ± 0.51^*∗*c^	1.90 ± 0.72^*∗*c^
intradermal Microdroplet	3.50 ± 0.51	1.20 ± 0.41^*∗*c^	0.70 ± 0.47^*∗*c^	2.10 ± 0.72^*∗*c^
nanomicroneedles	3.50 ± 0.52	2.05 ± 0.39^*∗*ab^	1.65 ± 0.49^*∗*ab^	2.9 ± 0.72^*∗*ab^

*F* value	0.257	34.911	31.298	10.857
*P* value	>0.05	<0.05	<0.05	<0.05

Note: ^*∗*^*P* < 0.05 compared with baseline; ^a^*P* < 0.05 compared with intramuscular injections group at the same time point; ^b^*P* < 0.05 compared with intradermal microdroplet group at the same time point; ^c^*P* < 0.05 compared with nanomicroneedle group at the same time point.

**Table 2:** Comparison of clinical effect on static wrinkles before and after treatment ($\overset{-}{x}\pm S$).

Group	Baseline	1 week	4 weeks	12 weeks
intramuscular injections	1.90 ± 0.55	1.75 ± 0.55^bc^	1.75 ± 0.55^bc^	1.80 ± 0.52
intradermal Microdroplet	1.90 ± 0.72	0.90 ± 0.55^*∗*a^	0.65 ± 0.49^*∗*a^	1.60 ± 0.50^*∗*^
nanomicroneedles	2.00 ± 0.65	1.10 ± 0.64^*∗*a^	0.85 ± 0.49^*∗*a^	1.80 ± 0.41^*∗*^

*F* value	0.257	11.636	26.357	1.152
*P* value	>0.05	<0.05	<0.05	>0.05

Note: ^*∗*^*P* < 0.05 compared with baseline; ^a^*P* < 0.05 compared with intramuscular injections group at the same time point; ^b^*P* < 0.05 compared with intradermal microdroplet group at the same time point; ^c^*P* < 0.05 compared with nanomicroneedle group at the same time point.

**Table 3:** Comparison of Subjective Satisfaction Scale after treatment ($\overset{-}{x}\pm S$).

Group	1 week	4 weeks	12 weeks
intramuscular injections	2.5 ± 0.53	3.1 ± 0.74	2.4 ± 0.52
intradermal Microdroplet	3.1 ± 0.74	3.3 ± 0.67	2.7 ± 0.67
nanomicroneedles	2.8 ± 0.63	2.8 ± 0.63	2.2 ± 0.42

*F* value	2.209	1.357	2.111
*P* value	>0.05	>0.05	>0.05

Note: ^*∗*^*P* < 0.05 compared with baseline; ^a^*P* < 0.05 compared with intramuscular injections group at the same time point; ^b^*P* < 0.05 compared with intradermal Microdroplet group at the same time point; ^c^*P* < 0.05 compared with nanomicroneedle group at the same time point.

**Table 4:** Comparison of adverse events after treatment ($\overset{-}{x}\pm S$).

Group	Pain score	erythema and edema score
intramuscular injections	7.3 ± 1.70^c^	2.4 ± 0.84^bc^
intradermal Microdroplet	7.4 ± 1.58^c^	1.7 ± 0.82^a^
nanomicroneedles	3.4 ± 1.65^ab^	1.4 ± 0.52^a^

*F* value	19.272	4.772
*P* value	<0.05	<0.05

Note: ^*∗*^*P* < 0.05 compared with baseline; ^a^*P* < 0.05 compared with intramuscular injections group at the same time point; ^b^*P* < 0.05 compared with intradermal Microdroplet group at the same time point; ^c^*P* < 0.05 compared with nanomicroneedle group at the same time point.

**Table 5:** Comparison of the skin elasticity before and after treatment ($\overset{-}{x}\pm S$).

Group	Baseline	1 week	4 weeks	12 weeks
intramuscular injections	0.30 ± 0.03	0.39 ± 0.03^*∗*^	0.44 ± 0.03^*∗*bc^	0.47 ± 0.08^*∗*bc^
intradermal microdroplet	0.32 ± 0.03	0.41 ± 0.04^*∗*^	0.49 ± 0.04^*∗*a^	0.56 ± 0.03^*∗*ac^
nanomicroneedles	0.32 ± 0.04	0.41 ± 0.03^*∗*^	0.50 ± 0.05^*∗*a^	0.41 ± 0.05^*∗*ab^

*F* value	1.891	2.28	10.796	33.558
*P* value	>0.05	>0.05	<0.05	<0.05

Note: ^*∗*^*P* < 0.05 compared with baseline; ^a^*P* < 0.05 compared with intramuscular injections group at the same time point; ^b^*P* < 0.05 compared with intradermal microdroplet group at the same time point; ^c^*P* < 0.05 compared with nanomicroneedle group at the same time point.

**Table 6:** Comparison of the skin hydration before and after treatment ($\overset{-}{x}\pm S$).

Group	Baseline	1 week	4 weeks	12 weeks
intramuscular injections	0.15 ± 0.04	0.20 ± 0.04^*∗*bc^	0.24 ± 0.03^*∗*bc^	0.22 ± 0.05^*∗*b^
intradermal microdroplet	0.18 ± 0.05	0.24 ± 0.04^*∗*a^	0.28 ± 0.03^*∗*a^	0.27 ± 0.08^*∗*ac^
nanomicroneedles	0.15 ± 0.04	0.23 ± 0.03^*∗*a^	0.28 ± 0.02^*∗*a^	0.20 ± 0.06^*∗*b^

*F* value	2.669	4.817	11.544	5.654
*P* value	>0.05	<0.05	<0.05	<0.05

Note: ^*∗*^*P* < 0.05 compared with baseline; ^a^*P* < 0.05 compared with intramuscular injections group at the same time point; ^b^*P* < 0.05 compared with intradermal Microdroplet group at the same time point; ^c^*P* < 0.05 compared with nanomicroneedle group at the same time point.

**Table 7:** Comparison of the skin collagen I content before and after treatment ($\overset{-}{x}\pm S$).

Group	Baseline	1 week	4 weeks	12 weeks
intramuscular injections	0.50 ± 0.08	0.59 ± 0.07^*∗*^	0.62 ± 0.06^*∗*bc^	0.64 ± 0.04^*∗*bc^
intradermal microdroplet	0.50 ± 0.06	0.60 ± 0.07^*∗*^	0.67 ± 0.05^*∗*a^	0.71 ± 0.03^*∗*ac^
nanomicroneedles	0.54 ± 0.08	0.63 ± 0.08^*∗*^	0.68 ± 0.08^*∗*a^	0.58 ± 0.08^*∗*ab^

*F* value	2.068	1.873	5.205	26.211
*P* value	>0.05	>0.05	<0.05	<0.05

Note: ^*∗*^*P* < 0.05 compared with baseline; ^a^*P* < 0.05 compared with intramuscular injections group at the same time point; ^b^*P* < 0.05 compared with intradermal microdroplet group at the same time point; ^c^*P* < 0.05 compared with nanomicroneedle group at the same time point.

**Table 8:** Comparison of the skin sebum content before and after treatment ($\overset{-}{x}\pm S$).

Group	Baseline	1 week	4 weeks	12 weeks
intramuscular injections	0.33 ± 0.03	0.28 ± 0.06	0.29 ± 0.05	0.30 ± 0.05
intradermal microdroplet	0.31 ± 0.04	0.26 ± 0.05	0.28 ± 0.06	0.28 ± 0.06
nanomicroneedles	0.32 ± 0.04	0.28 ± 0.06	0.27 ± 0.06	0.31 ± 0.03

*F* value	0.774	0.327	0.514	2.845
*P* value	>0.05	>0.05	>0.05	>0.05

Note: ^*∗*^*P* < 0.05 compared with baseline; ^a^*P* < 0.05 compared with intramuscular injections group at the same time point; ^b^*P* < 0.05 compared with intradermal microdroplet group at the same time point; ^c^*P* < 0.05 compared with nanomicroneedle group at the same time point.

## Data Availability

The data that support the findings of this study are available from Nanjing Medical University but restrictions apply to the availability of these data, which were used under license for the current study, and so are not publicly available. Data are however available from the corresponding author upon reasonable request and with permission of Nanjing Medical University.
